# Voiding function improves under long-term testosterone treatment (TTh) in hypogonadal men, independent of prostate size

**DOI:** 10.1007/s11255-023-03602-4

**Published:** 2023-05-06

**Authors:** Aksam Yassin, Mustafa Alwani, Raed M. Al-Zoubi, Omar M. Aboumarzouk, Raidh Talib, Joanne Nettleship, Daniel Kelly, Bassam Albaba

**Affiliations:** 1 Surgical Research Section, Department of Surgery, Hamad Medical Corporation and Men’s Health, Doha, Qatar; 2 Center of Medicine and Health Sciences, Dresden International University, Dresden, Germany; 3 Weill Cornell School of Medicine, Urology, Doha, Qatar; 4 Department of Biomedical Sciences, QU-Health, College of Health Sciences, Qatar University, 2713 Doha, Qatar; 5 Department of Chemistry, Jordan University of Science and Technology, PO Box 3030, Irbid, 22110 Jordan; 6 College of Medicine, Qatar University, Doha, Qatar; 7 School of Medicine, Dentistry and Nursing, The University of Glasgow, Glasgow, UK; 8 Biomolecular Research Centre, Sheffield Hallam University, Sheffield, UK; 9 Department of Oncology and Metabolism, Medical School, University of Sheffield, Sheffield, UK

**Keywords:** Hypogonadism, Testosterone, LUTS, Benign prostate hyperplasia, Prostate cancer

## Abstract

**Background:**

Functional hypogonadism is a condition in which some, but not all, older men have low testosterone levels. Rather than chronological age per se, the causality of hypogonadism includes obesity and impaired general health (e.g., metabolic syndrome). An association between testosterone deficiency and lower urinary tract symptoms (LUTS) has been reported, yet due to prostate safety concerns, men with severe LUTS (IPSS score > 19) have invariably been excluded from entering testosterone trials. Irrespective, exogenous testosterone has not been demonstrated to cause de novo or worsen mild to moderate LUTS.

**Objective:**

This study investigated whether long-term testosterone therapy (TTh) could have a protective effect on improving the symptoms of LUTS in hypogonadal men. However, the exact mechanism by which testosterone exerts is beneficial effect remains uncertain.

**Patients and methods:**

In this study 321 hypogonadal patients with an average age of 58.9 ± 9.52 years received testosterone undecanoate in 12-week intervals for 12 years. One hundred and forty-seven of these males had the testosterone treatment interrupted for a mean of 16.9 months before it was resumed. Total testosterone, International Prostate Symptom Scale (IPSS), post-voiding residual bladder volume and aging male symptoms (AMS) were measured over the study period.

**Results:**

Prior to TTh interruption, it was observed that testosterone stimulation improved the men’s IPSS, AMS and post-voiding residual bladder volume, while their prostate volume significantly increased. During the TTh interruption, there was a significant worsening in these parameters, although the increase in prostate volume continued. When TTh was resumed, these effects were reversed, implying that hypogonadism may require lifelong treatment.

## Introduction

Functional hypogonadism is characterized by low serum levels of testosterone and associated symptoms in males, demonstrating a higher prevalence as men age [1]. Low testosterone may lead to, and be exacerbated by, concomitant diseases such as metabolic syndrome (MetS), type 2 diabetes (T2D) and obesity, which tend to develop through age [2]. Low testosterone levels affect approximately 20% of men over the age of 60 years, 30% over 70 years and 50% over 80 years [3] and the associations between functional hypogonadism and its clinical features (including absence or regression of secondary sex characteristics, insulin resistance or T2D, hypertension, dyslipidemia, anemia, muscle wasting, reduced bone mass or bone mineral density, oligospermia, decreased libido, decreased sexual function and abdominal adiposity) and comorbidities leads to a lower quality of life (QoL) and often increased mortality [4]. Considering this, some studies have shown that testosterone therapy (TTh) has great therapeutic potential due to the favorable effects of testosterone on the comorbidities associated with androgenic deficiency [2]. However, there have been limited studies into the long-term effects of TTh and whether lifelong treatment is required.

Erectile dysfunction (ED) and lower urinary tract symptoms (LUTS) are significant contributors to age-related QoL in men and are both associated with several features of MetS, including obesity in epidemiological studies [5–7]. LUTS are often regarded as a hallmark of benign prostatic hyperplasia (BPH) with an increased incidence as men age [8]. Some studies have identified a correlation between functional hypogonadism and BPH whereby men may have significantly larger prostate volumes (PV) (> 31 mL) [3, 9] compared to men with normal testosterone levels [3]. Furthermore, a study by Schatzl et al. reported that approximately 20% of elderly men with LUTS had hypogonadism [10]. Reducing obesity as part of lifestyle intervention has been shown in some studies to lead to improvements in LUTS [11, 12] but not all [13, 14], and weight loss can result in modest increases in serum testosterone levels [15]. While the mechanisms underlying this potential inter-relationship between testosterone, obesity and LUTS are not known currently, evidence from some studies indicates that TTh may be a useful therapy for improving metabolic and urinary symptoms as well as comorbidities of late-onset hypogonadism (LOH) [16–18].

One of the concerns regarding TTh in elderly men remains increasing prostate volume and worsening urinary function parameters. Indeed, it is acknowledged that the prostate is an androgen-dependent organ that requires testosterone for growth and development, thus raising concerns about the potential risk of increased PV and therefore worsening of the associated risks (including LUTS and prostate cancer) if men are given TTh [19]. A controlled cross-sectional study conducted by Behre et al. (1994) concluded that TTh in hypogonadal men resulted in a significant increase in PV, comparable to age-matched normal men whereas hypogonadal men without TTh had lower PV [20]. This suggested that TTh resulted in a modest but significant increase in PV, however, still remained within normal limits. In contrast to this, a Chinese study showed after age adjustment, the rate of PV growth in aging patients with low testosterone was significantly greater than the normal testosterone level group, after 4 years [3]. In addition, a longitudinal study found that hypogonadal men who received TTh had a 12% increase in PV size on average [21]. However, another placebo-controlled study of hypogonadal males on TTh found no significant differences in PV between TTh-treated men and those on placebo [22]. Despite a potential testosterone-induced PV increase, current research suggests that TTh does not increase the risk of developing prostate cancer (PCa) [23–25] and can even protect against prostatic carcinoma in castration-resistant prostate cancer patients [26, 27] and men with advanced disease, namely, biochemical recurrence or metastatic PCa [28].

The International Prostate Symptom Score (IPSS) is used to assess the severity of LUTS and its related symptoms (for both irritative and obstructive symptoms) which result in a lower QoL; symptoms can include voiding and obstruction (hesitancy, poor/intermittent stream, straining, feeling of incomplete bladder emptying) as well as storage or irritative symptoms (frequency, incontinence and nocturia) [29]. In a Japanese population study of men with moderate to severe LUTS, TTh resulted in clinically significant improvements in the total IPSS score and the storage symptom score, but the voiding symptom score was not statistically improved after treatment [30]. In a meta-analysis of 14 clinical trials of TTh for hypogonadal men, the change in IPSS was similar among men receiving testosterone versus placebo, suggesting that TTh treatment does not worsen LUTS among men with hypogonadism [31]. The mean follow-up time for the studies included in this meta-analysis was 34.4 months and may indicate that long-term treatment is required for significant improvements in LUTS. Few studies have directly investigated the role of long-term TTh in hypogonadal males on the symptoms of LUTS. The protective role of testosterone and its improvements in LUTS in hypogonadal males were noted in our 2014 study [16]. The weight, waist circumference and BMI of the males also improved, further decreasing the risk for LUTS. Indeed, we have demonstrated that TTh interruption, and consequential reduced total testosterone levels, results in worsening of symptoms including obesity parameters, aging male symptoms (AMS), IPSS, residual voiding volume and bladder wall thickness, erectile function and prostate-specific antigen (PSA), while prostate volume remained unchanged until treatment resumed whereby these effects were reversed [32]. This suggests that hypogonadism may require lifelong TTh. In this retrospective registry study, we aimed to assess the long-term effects of testosterone treatment on the symptoms of LUTS (assessed by IPSS, AMS, post-voiding residual bladder volume, PV) in a cohort of 321 hypogonadal males with an average age of 58.9 ± 9.52 years in a urological setting.

## Patients and methods

In a population-based single-center, prospective, cumulative registry study, 321 hypogonadal men with a mean age of 58.9 ± 9.52 years (age range: 19–84 years), a baseline total testosterone level under 12.1 nmol/L on 2 morning blood samples and with clinical symptoms of hypogonadism along with absence of contraindication received 1000 mg injections of long-acting testosterone undecanoate (TU) (Nebido®, Bayer Pharma, Berlin, Germany) for 12 years in 12-week intervals. As assessed by the Aging Males’ Symptoms (AMS) scale, patients had at least moderate symptoms of hypogonadism with a baseline total testosterone (TT) of ≤ 3.50 ng/mL (≤ 12.1 nmol/L). Patients had documented ED for ≥ 6 months, established using the international definition for ED, the International Index of Erectile Function (IIEF). Patients with clinically significant findings on physical examination or presence of known clinically significant diseases that would prejudice the completion of the study or contraindicate testosterone administration were excluded from the study. Patients with severe diabetes mellitus, International Prostate Symptom Score (IPSS) > 18, prostatitis, hyperprolactinaemia (> 20 ng/mL) or cardiovascular events within the last 6 months were also excluded from this study, as were patients with obstruction due to BPH with residual urine higher than 40 mL. The institution received approval from the ethics committee in accordance with German Ärztekammer (German Medical Association) regulations. Patients were enrolled once they had signed an informed written consent and all data was treated with confidentiality (Table 1).

**Table 1:** Baseline characteristics of the study population

Baseline patient characteristics	
*N*	321
Mean age (years)	59 ± 9.5
Follow-up (years)	8.3 ± 3.5
Testosterone (nmol/L)	7.7 ± 2.1
PSA (ng/mL)	0.95 ± 0.61
Waist circumference (cm)	107.5 ± 10
Weight (kg)	99 ± 13
BMI (kg/m^2^)	31.5 ± 4.3
GFR (mL/min/1.73 m^2^)	87.0 ± 12.8
Baseline comorbidities	
Hypertension	221 (68.8%)
Hypothyreosis	5 (1.6%)
Hyperthyreosis	0 (0%)
Type II diabetes	94 (29.3%)
HbA1c (for patients with type II diabetes)	7.9 ± 1.0%

As previously described [2, 32], the cohort received TTh for a mean duration of 65.5 ± 14.1 months before being temporarily interrupted for 147 men for a mean 16.9 ± 3.3 months. The interruption was due to reimbursement issues in 140 men and 7 men were diagnosed with prostate cancer and had a mean withdrawal of 16.9 months. All men resumed their treatment thereafter. The remaining 174 patients were treated without interruption. All injections were administered and documented during the clinical visit; treatment compliance was 100%. Three men dropped out of the study for unknown reasons. Throughout the trial, blood samples were taken from the participants at every other visit as were prostate volume (transrectal ultrasound) and post-voiding residual bladder volume by ultrasonography. Treatment-emergent adverse events were monitored throughout the study. Physical examination, digital rectal examination (DRE) serum prostate-specific antigen (PSA) levels were also measured at each 3-month period. International Prostate Symptom Scores (IPSS) and Aging Males’ Symptoms (AMS) questionnaires were assessed at each treatment visit. An independent commercial laboratory measured the total testosterone in serum samples. Alpha blockers and 5α-reductase inhibitors were used in 54% of the patients. Medications were not altered during the study. No acute urinary retention or prostate surgery was reported during the follow-up time.

### Statistical analysis

Patient data were averaged over the course of their study participation and expressed as mean values with standard deviations at each time point. Before the first TU injection, the baseline parameter values were recorded. The continuous variable change from baseline and change from the prior year were compared using analysis of variance (ANOVA). The Statistical Package for Social Sciences v.18 was used for statistical analysis (SPSS Inc., Chicago, USA) and GraphPad Prism version 8.4.3 (GraphPad Software, La Jolla, CA, USA). A value of *p* < 0.05 was considered significant.

## Results

**Fig. 1:**
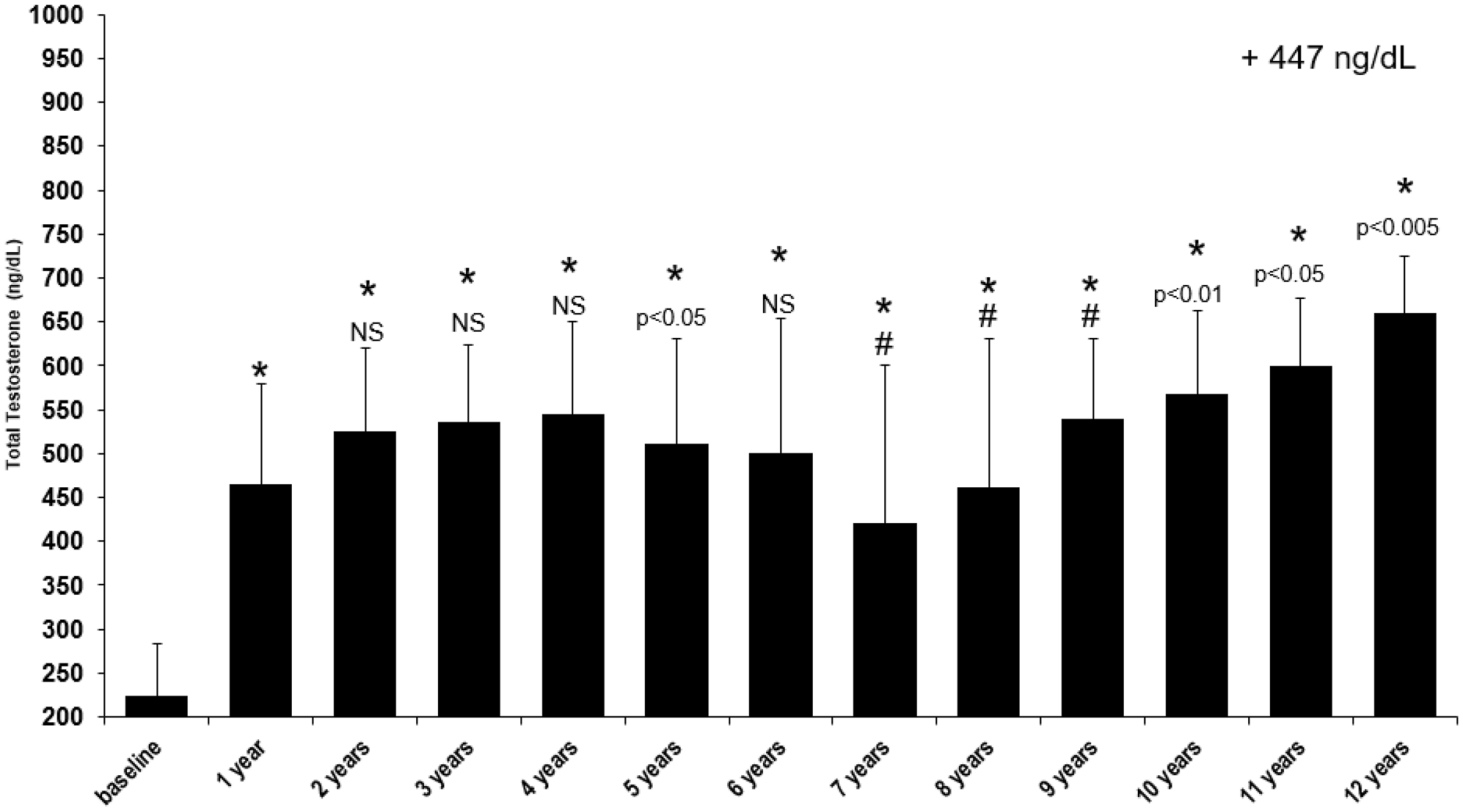
Long-term effect of testosterone treatment on total testosterone (TT) levels (ng/dL) in hypogonadal males. *p < 0.0001 vs. baseline; #*p* < 0.0001 vs. previous year; all other *p* values compared to previous year

Prior to TTh, total testosterone (TT) levels in the hypogonadal men were measured at a mean 223.22 ± 62 ng/dL and within the first year this significantly increased twofold where it then reached a steady state (years 2 and 4) (Fig. 1). TT levels saw a temporary reduction in the group of patients that had their treatment interrupted (data reported elsewhere [32]) which influenced the mean TT levels for the cohort by reducing levels between 6 and 8 years. Upon retreatment, TT again increased where it was at its greatest level in the final year (year 12) with a difference of 447 ng/dL compared to the baseline (p < 0.0001 vs. baseline).

**Fig. 2:**
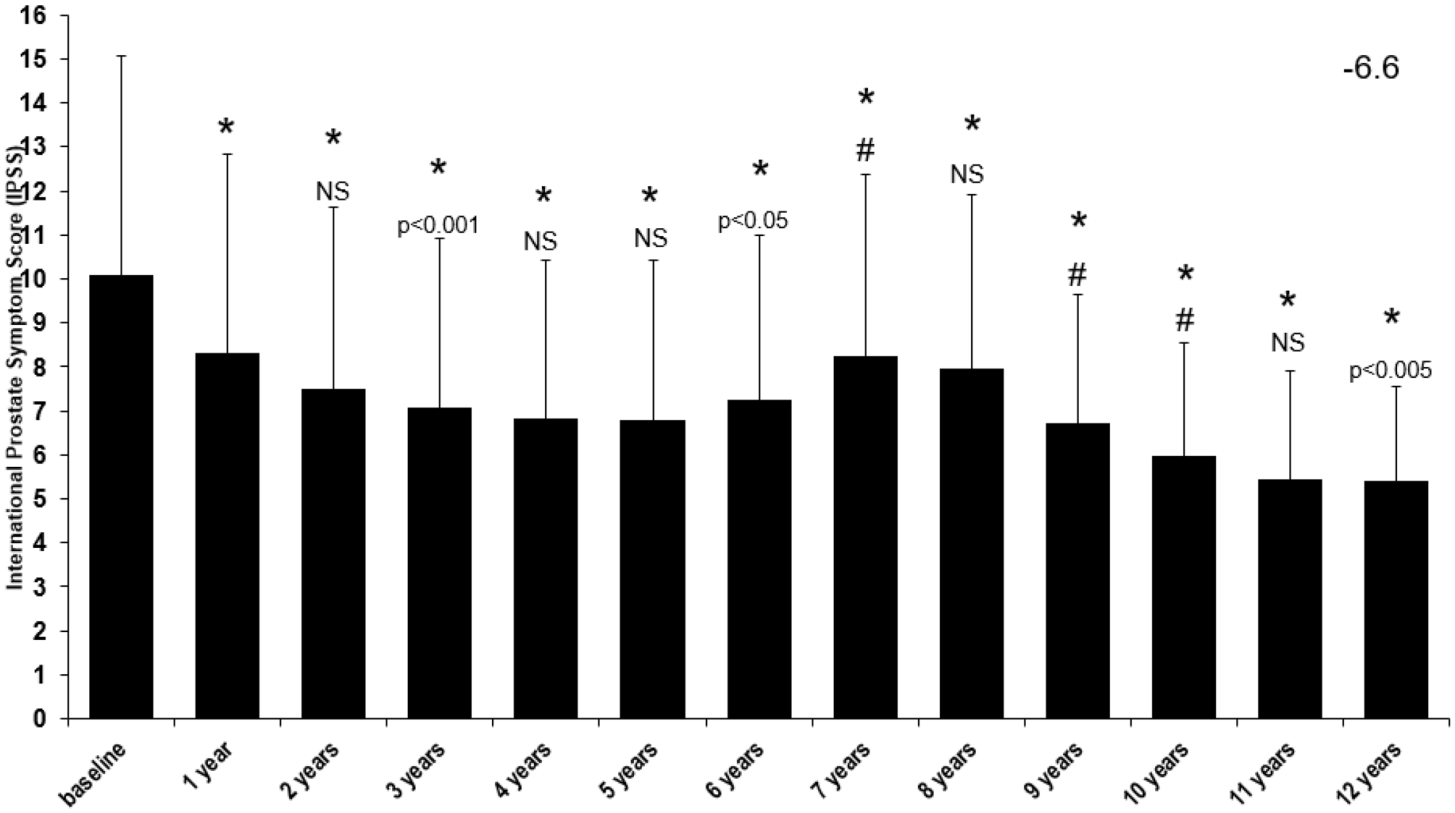
Long-term effect of testosterone treatment on International Prostate Symptom Score (IPSS) in hypogonadal males. *p < 0.0001 vs. baseline; #*p* < 0.0001 vs. previous year; all other p values compared to previous year

TTh concurrently reduced the hypogonadal males’ IPSS score (Fig. 2). Initially, it had a mean value of 10.1 ± 5.0 and then had a steady decline until the treatment was interrupted, visualized between years 5 and 8 where it then increased from 6.79 ± 3.63 (year 5) to 8.24 ± 4.15 (year 7). A gradual decline was then visualized again between 8 and 12 years of TTh with the lowest IPSS score being in the final year (5.38 ± 2.17) (*p* < 0.0001 vs. baseline).

**Fig. 3:**
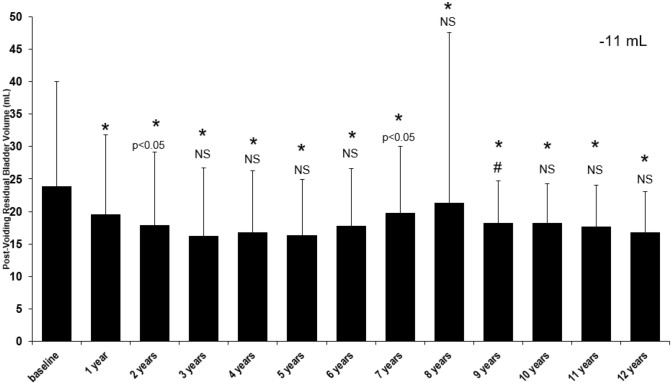
Long-term effect of testosterone treatment on post-voiding residual bladder volume (mL) in hypogonadal males. *p < 0.0001 vs. baseline; #*p* < 0.0001 vs. previous year; all other *p* values compared to previous year

During the TTh, post-voiding residual bladder volume was measured (Fig. 3). It was observed that there was a decrease from 23.8 ± 16.2 mL to 16.7 ± 6.4 mL (*p* < 0.0001 vs. baseline) with a temporary increase in years 6–8.

**Fig. 4:**
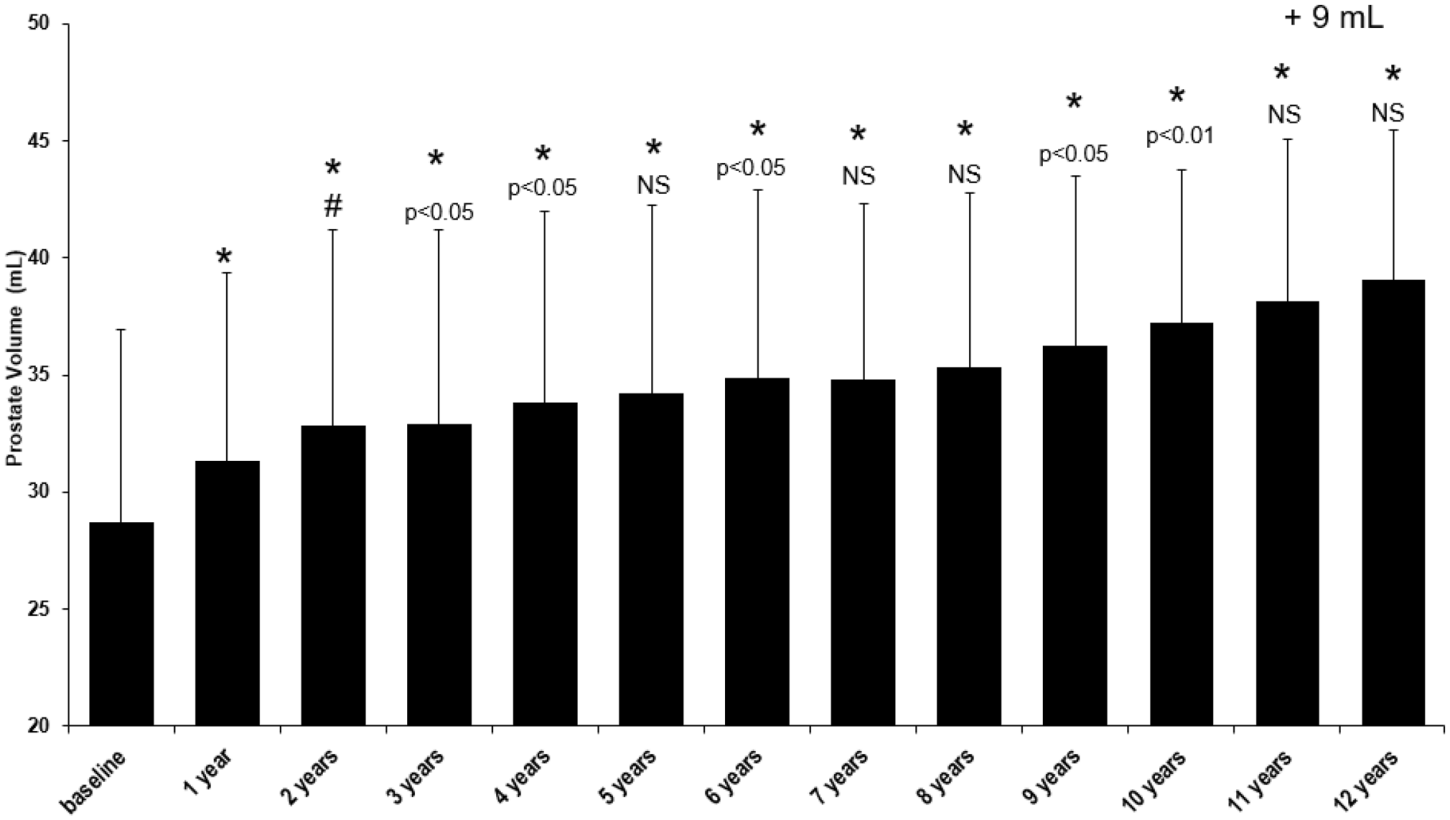
Long-term effect of testosterone treatment on prostate volume (mL) in hypogonadal males. *p < 0.0001 vs. baseline; #*p* < 0.0001 vs. previous year; all other *p* values compared to previous year

Throughout the study, prostate volume was also measured (Fig. 4). Initially, the mean PV was 28.7 ± 8.3 mL and following TTh, there was a significant increase all the way through to year 12 with an average increase of + 10.3 mL to 39.0 ± 6.4 mL (*p* < 0.0001 vs. baseline) without deviation from the trend during years 6–8, when TTh was interrupted for 147 men.

**Fig. 5:**
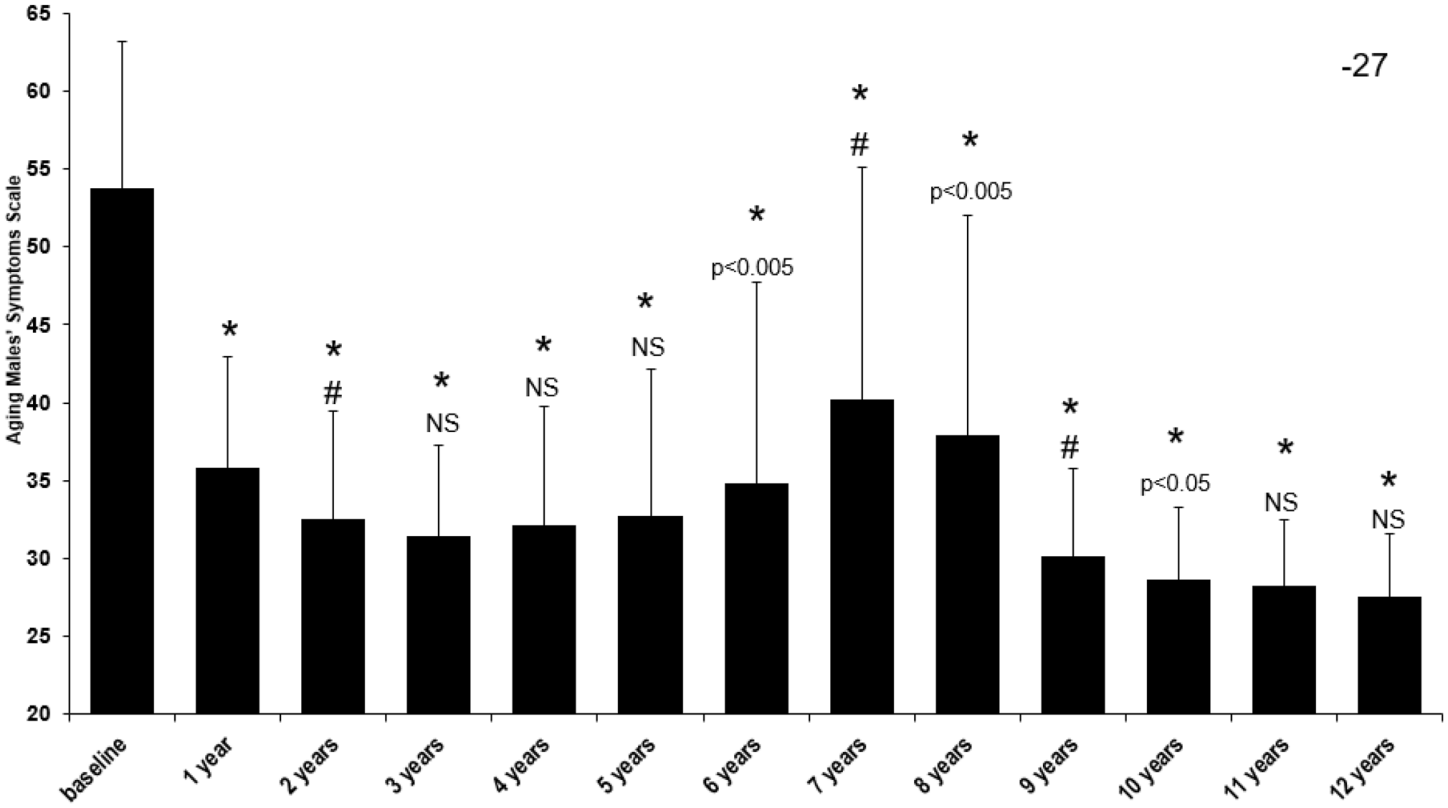
Long-term effect of testosterone treatment on Aging Males’ Symptom Scale in hypogonadal males. *p < 0.0001 vs. baseline; #*p* < 0.0001 vs. previous year; all other *p* values compared to previous year

To measure the health-related quality of life, the Aging Males’ Symptoms Scale was utilized. Figure 5 indicated there was a significant improvement from the baseline with a decrease from 53.7 ± 9.5 to 27.5 ± 4.0 (*p* < 0.0001 vs. baseline) with a temporary increase in years 6–8.

## Discussion

Hypogonadism is commonly associated with concomitant diseases including MetS, obesity, T2D, ED and LUTS. The therapeutic treatment of hypogonadism with testosterone supplementation has shown a protective role in the development and treatment of these diseases, with many studies observing improvements in the parameters of obesity, body composition, glycemic control, ED and QoL [2, 4, 15, 17, 33]. However, the use and safety of long-term TTh is still widely debated due to insufficient randomized controlled clinical trial data and some conflicting studies.

In men, LUTS are often concurrent with BPH, and a number of therapeutic treatments exist which can reduce prostate volume by approximately 25% [34, 35]. In particular, 5-alpha reductase inhibitors (5ARIs) result in an 85% reduction in prostatic dihydrotestosterone and are a common, effective therapy option for men with BPH-related LUTS who have demonstrable prostate enlargement. Treated patients demonstrated decreased prostate volume, improved urinary symptoms, and decreased risk of urinary retention; however, several sexual side effects were present including ED, gynecomastia, decreased libido and decrease in ejaculate volume indicating a lower QoL [36]. The findings of this present study are unique, in that long-term TTh in hypogonadal men improved voiding function, symptoms of LUTS and QoL, independent of prostate volume.

Several studies examining the relationship between prostatic size and LUTS in hypogonadal men receiving TTh indicated no significant increase in prostate size or LUTS with testosterone replacement [22, 31]. Furthermore, there is evidence that TTh can improve LUTS, as Saad et al. found significant improvement in IPSS scores in 28 men with sexual dysfunction and metabolic syndrome treated for 12 months with long-acting testosterone undecanoate [37]. This is supported by our previous study showing significant IPSS score reduction in 261 hypogonadal men with MetS after over 5 years TTh [38, 39]. As with the present study, Karazindiyanoğlu and Cayan showed that 25 hypogonadal men with sexual dysfunction were able to improve LUTS (indicated by IPSS, AMS, IIEF-5), as well as bladder function, following 1 year of TTh compared to placebo-treated controls [40]. Of note, PV significantly increased from 25.24 ± 6.74 mL to 28.8 ± 7.01 mL, although no significant changes in PSA and free PSA levels were shown [40]. Even while PV increased significantly during the present study, the lack of a placebo-controlled group makes it difficult to draw judgments about whether the increase in PV was above that of the normal aging population.

Contraindications for the use of TTh is the increased risk and causality of prostate carcinogenesis/androgen-dependent tumors due to potential PV increase, although increasing evidence suggests that testosterone does not increase PCa and may even be beneficial [41–43]. In a recent study, testosterone undecanoate improved urinary function and anthropometric parameters in 412 hypogonadal men and, alongside this, also discovered that TTh had no effect on the prostate parameters with incident prostate cancer reduced in the testosterone-treated group compared to the control group [44]. A randomized, double-blind, placebo-controlled study of 44 hypogonadal men failed to demonstrate a higher risk of prostate cancer in men on TTh, as there was no treatment-related change in prostate histology, tissue biomarkers, gene expression, or cancer incidence or severity (but minor PV increase) when they received 150 mg of testosterone enanthate or matching placebo for a duration of 6 months [45]. Despite this, the relationship between testosterone and PCa remains complex, and recommendations suggest not to use TTh where there is suspected or confirmed prostate malignancies [46, 47]. These proposed risks require further investigation in long-term controlled trials, and in the clinic they need to be weighed against the potential improved health-related QoL. Contrawise, with growing evidence that lower, not higher, testosterone levels trigger the development of PCa and BPH through androgen receptor over-expression, a paradigm shift in altering the present approach to diagnosing and treating men with hypogonadism is advocated to improve men’s health [48]. Regardless, all patients receiving TTh require close monitoring for safety.

LUTS and BPH negatively impact men’s health-related QoL [49]. The treatment of hypogonadal males with TTh has been previously shown to improve QoL, ED and LUTS [16, 32, 44] and is further observed in this present study. The improvement in QoL of hypogonadal men receiving TTh may be due in part to the relief of LUTS symptoms (e.g., voiding dysfunction) but it is also likely due to improvements in the associated effects on sexual function (ED, ejaculatory dysfunction, libido) [50], as seen in a meta-analysis where TTh provided significant improvement in all aspects of sexual function in hypogonadal men [51].

The mechanistic relationship between testosterone and LUTS is complex and remains poorly understood, although a pathophysiological link between components of MetS and LUTS has been proposed. Studies have revealed a possible link between insulin resistance (diabetes mellitus) and LUTS in BPH patients [52] and according to the findings of a meta-analysis, people with diabetes have more severe LUTS than individuals without diabetes, as determined by IPSS [53]. Furthermore, there is evidence strongly linking obesity and the incidence of LUTS/BPH, and the pathology of both obesity and MetS is underpinned by chronic inflammation which additionally plays a crucial role in BPH [54]. Therefore, the inter-relationship between diabetes, obesity, MetS and inflammation contribute to LUTS progression. Low testosterone in men is associated with these comorbidities and several studies have demonstrated the beneficial effects of TTh on insulin sensitivity, parameters of obesity and inflammation [33, 55–58]. Specifically, TTh reduced BMI, WC, HbA1c, and CRP in parallel to an improvement in LUTS in the same cohort of hypogonadal men as the present study over an 8-year follow-up [44]. Nitric oxide (NO) is a potent regulator of prostatic innervation and pelvic smooth muscle tone and is considered to play a role in LUTS. NO levels result in more severe LUTS [59]. Accordingly, phosphodiesterase-5 (PDE-5) inhibitors are effective in the treatment of BPH/LUTS as well as erectile function [60–62]. Evidence suggests that testosterone may improve LUTS, as well as erectile function and associated QoL at least partly via a mechanistic action on NOS and via NO signaling.

The current research has some limitations. Due to this being an observational study with no placebo-controlled group, the direct effects of treatment versus no treatment could not be evaluated, limiting the interpretation of the data. Additionally, TTh was interrupted in 147 males due to reimbursement issues and/or the diagnosis of prostate cancer. While this may skew the data presented, particularly for years 6–8, we have previously discovered that when TTh is resumed, testosterone levels return to pre-interruption levels [32]. As a result, the interruption is unlikely to have altered the circulation levels of hormones measured during the final 12-year follow-up. Indeed, all parameters measured in this study, except prostate volume, regressed in those patients in whom TTh was temporarily interrupted but started improving again after resuming TTh, suggesting a high degree of reversibility. The worsening after testosterone cessation was also observed in short-term studies [63, 64]. The study by Francomano et al. observed that in severely obese men, metabolic, fat but not lean mass, and blood pressure parameters were maintained for a relatively short duration, whereas cardiac and hormonal parameters returned to baseline post-TTh [33]. It is understood that the maintenance of some of the measures may occur over a short term, but may be gradually lost over longer durations. Notably, many of the positive effects of TTh, especially in relation to body composition and weight, can take between 6 and 12 months to manifest [17]. Therefore, the long-term duration of the data collected here is a strength of the present study.

In conclusion, this study suggests that long-term TTh can improve voiding function and alleviate symptoms of LUTS seemingly independently of prostate volume. These therapeutic benefits develop in parallel to improvements to QoL, as indicated by AMS. TTh may need to be continued indefinitely to retain the favorable effects, as we have previously observed in this cohort a worsening of parameters when TTh is discontinued and the sustained benefits over 12 years in the present study. While TTh may not increase prostate risk despite increases in PV, there is a need for large, placebo-controlled long-term outcome studies to validate current suggestions with more conclusive evidence.

## Data Availability

The data supporting this study's findings are available from the corresponding author upon reasonable request.

## Abbreviations

LUTS: Lower urinary tract symptoms
IPSS: International Prostate Symptom Score
TTh: Testosterone therapy
AMS: Aging male symptoms
MetS: Metabolic syndrome
T2D: Type 2 diabetes
QoL: Quality of life
ED: Erectile dysfunction
PV: Prostate volumes
LOH: Late-onset hypogonadism
PCa: Prostate cancer
PSA: Prostate-specific antigen
TU: Testosterone undecanoate
IIEF: International Index of Erectile Function
DRE: Digital rectal examination
TT: Total testosterone
BPH: Benign prostatic hyperplasia
BMI: Body mass index
WC: White blood cell count
HbA1c: Hemoglobin A1C
CRP: C-reactive protein
NOS: Nitrous oxide
NO: Nitric oxide
